# Romanian Translation and Cultural Adaptation of the Seizure Severity Questionnaire

**DOI:** 10.3390/neurolint16050076

**Published:** 2024-09-13

**Authors:** Ionut-Horia Cioriceanu, Dan-Alexandru Constantin, Bianca Zamfirescu, Petru Cezar Podasca, Luigi Geo Marceanu, Liliana Rogozea

**Affiliations:** 1 Department of Neurology, Clinical Hospital of Psychiatry and Neurology Brasov, 500123 Brasov, Romania; dr.cioriceanu@gmail.com; 2 Department of Fundamental, Prophylactic and Clinical Sciences, Faculty of Medicine, Transilvania University of Brasov, 500019 Brasov, Romania; r_liliana@yahoo.com; 3 Department of Social Work and Psychology, Dr. I.A. Sbarcea Clinical Hospital of Obstetrics and Gynecology Brasov, 500025 Brasov, Romania; bia.zupcau@gmail.com (B.Z.); cezarpodasca@yahoo.com (P.C.P.); 4 Department of Medical and Surgical Specialties, Faculty of Medicine, Transilvania University of Brasov, 500019 Brasov, Romania

**Keywords:** seizure severity, SSQ, epilepsy

## Abstract

The aim of this study was to report the translation into Romanian of the Seizure Severity Questionnaire (SSQ), an instrument for the evaluation of the frequency and severity of epileptic seizures, and the results of applying it to a group of patients with epilepsy evaluated at a hospital in Romania. Methods: Four translators were involved in obtaining conceptual analogies and the cultural importance of the translated notions. The final version was obtained for the Romanian population, with the same appearance as the original instrument. Sixty-seven patients with epilepsy completed the SSQ and the Patient-Weighted Quality of Life in Epilepsy Inventory—QOLIE-31-P. Results: Females had a lower mean SSQ total score (TS) and perceived seizures less seriously than men. Patients with epilepsy with aura had a higher mean SSQ TS, with a more severe seizure perception, compared to those without aura. According to the frequency of seizures, patients with epilepsy with rare seizures had the lowest mean SSQ total score (TS) compared to those with frequent seizures. Patients who were on monotherapy had a less severe perception of epileptic seizures compared to those who were treated with two or more antiepileptic drugs. All QOLIE-31-P domains and TS correlated statistically significantly with the SSQ TS. Conclusions: This study explored SSQ translation, evaluated preliminary results, and showed the correlation between seizure frequency and severity, clinical factors, and quality of life. This tool could be useful for measuring seizure severity in Romanian patients with epilepsy and conducting comparative studies.

## 1. Introduction

Epilepsy is a chronic neurological disease characterized by repeated and unprovoked seizures, caused by abnormal electrical activity in the brain [1]. With 50 million people affected worldwide and an estimated prevalence of 8.23 per 1000 people in Europe [2,3], epilepsy stands as one of the most prevalent neurological diseases. The consequences of epilepsy go beyond the manifestation of seizures, since the disorder may significantly interfere with multiple areas of the life of a person, including their cognitive abilities, emotional state, and social interactions. Despite the progress made in the field of therapy, a significant number of individuals with epilepsy still experience seizures or adverse reactions to medical therapies [4]. This highlights the imperative for continuous research and enhanced therapeutic alternatives. Epilepsy research in Romania has been part of a broader effort to improve the management and treatment of patients with epilepsy. Although epidemiologic studies are rare, they have revealed a prevalence rate comparable to that of other European countries [3]. 

The main objective of epilepsy treatment is to reduce or completely control seizures. It is well known that an increased frequency of epileptic seizures is associated with poorer quality of life (QOL) for people with epilepsy [5,6,7]. The assessment of the impact of seizures has conventionally focused on the frequency and types of seizures, but these classifications do not take into account particular aspects such as seizure manifestations and subsequent effects, falls, changes in consciousness, or the perception of seizure control in patients with epilepsy.

These concerns are important in patients in whom complete seizure remission cannot be achieved, and reducing seizure severity can be a key factor in improving QOL. However, validated and standardized seizure severity assessment tools are not available in Romanian, and there have been no studies conducted in our country to highlight the reliability of using these tools.

In the last four decades, a number of scales have been developed to assess seizure severity, such as the Veterans Administration Seizure Frequency and Severity Scale [8], the Chalfont Seizure Severity Scale [9] later renamed the National Hospital Seizure Severity Scale [10], the Liverpool Seizure Severity Scale [11], and the Hague Seizure Severity Scale [12], but limitations such as the complexity of reporting, inadequate weighting, a lack of distinction between seizure types, and a lack of confirmed sensitivity prevented their use in clinical practice and clinical trials [13].

The Seizure Severity Questionnaire (SSQ) was initiated in 1999 and underwent an initial validity and reliability assessment in 2002 [14]. Subsequently, the instrument has been increasingly used in clinical trials to assess seizure severity [15]. The evolution of tools for evaluating the severity of seizures has made significant progress since their inception, and there has been a noticeable shift in their usefulness. Thus, the performances of the instruments could be verified not just in controlled drug trials but also in real clinical environments [16]. Despite the diversity and disparities in the development process of these instruments, the SSQ has the capacity to complete its intended purpose. Following the publication of the SSQ, subsequent antiepileptic drug trials incorporated the tool as an element of the efficacy assessment, consequently adding the further benefit of detecting changes in the clinical state. Such studies have shown an improvement in the severity of seizures with the medications investigated and also an improved QOL [17]. Enhancements and perspectives gained from the limitations of the previous tools will enhance the usability and functionality of the new ones, confirmed by supplementary assessments to evaluate their sensitivity and responsiveness. One such proposed recommendation is to integrate electrical brain events from electroencephalogram reports with physical seizure occurrences to evaluate severity [18].

The primary objective of this study was to report the translation and cultural adaptation of the SSQ on a group of patients with epilepsy evaluated at a hospital in Romania. The secondary objective was to evaluate the relationship between the severity of epileptic seizures and the frequency of seizures in patients diagnosed with epilepsy. The hypothesis of the study is based on the assumption that there are statistical correlations between the total score of the SSQ, the domain scores, and the total score of the Patient-Weighted Quality of Life in Epilepsy Inventory—QOLIE-31-P (QOLIE-31-P), and that this instrument for measuring seizure severity may be relevant in its application to Romanian patients with epilepsy alongside the quality-of-life assessment questionnaire.

## 2. Materials and Methods

### 2.1. Translation

This study began with permission from the copyright holder and author of the questionnaire, Joyce Cramer, who provided us with the original English version so that we could begin the Romanian translation process. The translation was carried out by an authorized translation agency and completed with a report of the translation process.

Four translators with experience translating medical texts were involved. The project manager organized and distributed the English version, taking into account the following criteria for the selection of translators: experience in translation, authorization, care, and thoroughness of the document. In the first translation phase, two translators were selected—native Romanian speakers who know and speak English—one being a sworn translator and the other being an employee of the translation office. The third translator made a combination of the two translations, using in the final translation the terminology used by one as well as the other and mentioning the phrases that were better used in their own terms. Subsequently, the first two translators had the opportunity to analyze each other’s materials and discuss any difficulties they might have encountered. The fourth native Romanian-speaking translator with a high level of understanding and writing in English has been used to analyze the concepts and content and avoid inspiration from the original English material.

The aim was to obtain the conceptual analogy and cultural importance of translated notions and to ensure that the instrument is understandable for Romanian patients. After testing the final form on ten patients with epilepsy without cognitive impairment in a pilot study, it was determined that the terms were highly accepted. This resulted in the final version for the Romanian population, with the same appearance and images as the original instrument. A copy of the version, along with the information necessary to fulfill the translation requirements, was provided to Joyce Cramer for archiving and distribution to other researchers as needed.

### 2.2. Participants

This study included a cohort of sixty-seven patients between the ages of 18 and 79 who had been clearly confirmed to have epilepsy based on ILAE criteria [19]. It was conducted at the Clinical Hospital of Psychiatry and Neurology in Brasov, Romania, from February 2018 to August 2021. No specific selection was made; only patients with epilepsy and other progressive psychiatric or neurological diseases, severe physical pathologies, intellectual disabilities, and problems comprehending parts of the research tools were excluded.

### 2.3. Study Instruments

Two research instruments were used, the Patient-Weighted Quality of Life in Epilepsy Inventory—QOLIE-31-P [20]—and the Seizure Severity Questionnaire—SSQ.

The QOLIE-31-P is a QOL measure specific to epilepsy and intended for use for individuals 18 years of age and older. The 38 items in the assessment contain 7 domains: energy, mood, daily activities, cognition, medication effects, seizure worry, and overall QOL. Every domain scale contains a single question that serves as a weighting system to determine the respondent’s level of distress regarding concerns related to the disease. Higher scores are indicative of greater QOL, and the scoring process complies with the QOLIE-31-P scoring guide. The scores range from 0 to 100. Before using the inventory, we requested and received approval. We used the Romanian version of the QOLIE-31-P, which was provided and returned by Cramer J. for our study. Clinical studies involving patients with epilepsy in Romania have previously used the QOLIE-31-P [21,22].

The SSQ is designed as a self-report assessment instrument with 24 items and divides epileptic seizures into three phases: warning, ictal activity, and seizure recovery. The recovery phase is comprised of three separate aspects, specifically the cognitive, emotional, and physical dimensions of recovery, each of which is assessed in terms of frequency, severity, and feeling of bother. The scoring algorithm version 3 has been made available by the copyright holder along with the English version. The SSQ TS ranges from 0 to 7, with higher scores indicating a greater severity of seizures.

The SSQ, therefore, covers a patient-reported multidimensional assessment of seizure components, with a focus on the recovery period as the most problematic aspect of seizures [20]. In this research, we have used the version translated into Romanian according to the requirements imposed on the request for permission of use and explained previously.

### 2.4. Procedures

Patients with good language, writing, and comprehension skills and a confirmed diagnosis of epilepsy based on clinical, electrophysiological, and imaging examinations who presented for investigation by video-electroencephalography (video-EEG) were invited to participate in the study, and the purpose and procedure were explained to them. After being provided with the possibility to ask about the research, it was clearly explained that a refusal to participate would not cause any inconvenience. Those who expressed their agreement proceeded to sign consent forms for both participation and dissemination of their findings.

The QOLIE-31-P was initially administered, followed by the SSQ. Sixty-seven patients completed both questionnaires in their entirety. Questionnaires have been assigned numbers to achieve anonymity. The localization of epileptiform activity has been studied using digital video-EEG (Natus, Middleton, WI, USA). Socio-demographic data collected were age, sex, residence, marital status, education, and socio-professional status, and clinical data were duration of illness, frequency of epileptic seizures, etiology of epilepsy, number of antiepileptic drugs, and presence of aura.

### 2.5. Data Analysis

The analysis of the data was performed using GraphPad Prism version 10.2.1 software (GraphPad Software, Boston, MA, USA). Absolute values and percentages were utilized for categorical variables, and statistical processing involved measuring mean scores and standard deviations (SDs) for the variables. Epileptic seizures were divided by frequency into one per month, two or more per month, and between one and six per year. A *t*-test was used to test for associations between the SSQ total score (TS) and the QOLIE-31-P total and domain scores and between the SSQ TS and clinical characteristics. The level of statistical significance was set by a *p*-value < 0.05 and a 95% confidence interval. 

### 2.6. Ethical Aspects

The Research and Ethics Committee of Transilvania University of Brasov, by decision No. 1.1/21.05.2018, as well as the management of the Clinical Hospital of Psychiatry and Neurology Brasov, Romania, by decision No. 1121/30.01.2018, approved the research, which complied with the Code of Ethics of the World Medical Association.

## 3. Results

Sixty-seven patients were eligible, and 56.7% (*n* = 38) were female. The mean (SD) age was 44.07 (±15.06) years, with a mean (SD) disease duration of 14.39 (±15.74) years. Seventy-four-point-six percent (*n* = 50) of participants lived in urban areas, 59.7% (*n* = 40) were married, 39% (*n* = 26) were employed, and 46.3% (*n* = 31) had a high formal level of education. Patients included in this sample did not encounter any difficulty in understanding, following, or completing the two questionnaires. The time taken to answer the questions was approximately 30 min. All participants had a confirmed diagnosis of epilepsy and had seizures in the last year. The majority of patients had structural epilepsy, with a frequency of one to six seizures per year and no aura at the onset.

Of the study participants, females had a lower mean SSQ TS and perceived seizures less seriously than men. Those who presented aura at the onset of seizures had a higher mean SSQ TS, with a more severe seizure perception, compared to those without aura. According to the frequency of seizures, patients with epilepsy with rare seizures, between one and six per year, had the lowest mean SSQ TS compared to those with frequent seizures. Patients with two or more seizures per month had the highest mean SSQ TS. According to the etiology of the disease, we did not identify a statistically significant correlation with the SSQ TS of those with genetic epilepsy, but in those with structural etiology, the mean SSQ TSs were higher compared to those with unknown etiology. By the number of antiepileptic drugs, patients who were on monotherapy had a less severe perception of epileptic seizures compared to those who were treated with two or more antiepileptic drugs (AEDs); the least-severe perception of seizure occurred in those who did not receive treatment (Table 1).

The mean (±SD) SSQ TS was 3.114 (±1.477). All QOLIE-31-P domains correlated statistically significantly with the SSQ TS, as did the QOLIE-31-P TS (Table 2).

## 4. Discussion

Through this study, the translation from English to Romanian of SSQ and its application to a heterogeneous sample of patients with epilepsy were achieved. Patients who completed the full questionnaire did not encounter problems browsing or understanding it. The study showed that women had a better perception of the severity of seizures compared to men. This result is consistent with other research that has shown that men, by the nature of their occupation, can be more exposed to risk factors such as skull injuries or alcohol consumption [23]. Another study showed that women are more likely to hide epilepsy symptoms for socio-cultural reasons [24].

Respondents who had epileptic seizures with auras had a more severe perception of seizure severity. Aura is a subjective ictal phenomenon that can include different sensations and is a clinical sign of a seizure that may occur before the alteration of consciousness. It can often be recalled after a seizure. Studies that have investigated the influence of aura on the severity of seizures have contradictory results. Some studies suggest that the presence of an aura may increase the severity perceived by the patient about seizures, possibly due to anxiety or fear associated with the aura experience [25]. Other studies have shown no significant correlation between the presence of aura and the severity of seizures [26]. This relationship is complex and may depend on individual factors or mechanisms of secondary discharge propagation and the epileptogenic network [27]. More research is needed to understand this relationship.

The etiology of epilepsy is a major prognostic factor for the recurrence of seizures [28]. Focal epilepsies associated with structural brain abnormalities are less likely to go into remission than those occurring in patients with normal brain structures revealed by imaging. This study supports higher seizure severity in patients with structural epilepsy, which is similar to other research that has shown that patients with structural epilepsy were more likely to experience the worst outcome trajectory patterns compared to patients with another etiology. However, patients with structural epilepsy may have remission and, therefore, benign forms [29].

This study shows a correlation between seizure severity and the number of antiepileptic drugs. Participants in the study who were treated with two or more antiepileptic drugs perceived the severity of the seizures most seriously, compared to those under monotherapy or those who were not treated, contrary to the recommendations. However, this correlation may vary per individual and may not be direct. Patients treated with multiple antiepileptic drugs may experience more severe seizures due to treatment resistance or the type of epilepsy. In the case of other patients with epilepsy, the combination of antiepileptic drugs can effectively control the disease, leading to less severe seizures or even their absence [30,31]. Other factors that can influence the relationship between the number of antiepileptic drugs and the severity of seizures include the effectiveness of prescribed medications, the presence of comorbidities, the type of epilepsy, or individual responses to treatment [32]. It is important that doctors carefully evaluate each patient and adjust the treatment plan to achieve optimal seizure control and minimize side effects and possible drug interactions.

In this study, patients with fewer seizures were less likely to perceive seizures as severe than those with frequent seizures. Previous studies have shown that patients with epilepsy with frequent seizures experience a greater impact on QOL compared to those with rare seizures and controlled illnesses [33], and those with frequent and severe seizures experience a significant impairment of physical health, emotional well-being, and social relationships [34]. The results from this study may suggest that by reducing the frequency of seizures, the perception of their severity may be improved. The SSQ TS was correlated with the QOLIE-31-P scores, a questionnaire validated for the Romanian population and previously used in clinical trials. These results obtained on a smaller group of patients, which represent a limitation of the study, may be useful in applying the SSQ in future larger research on more patients and in different areas of the country.

One of the barriers to this study was the limited access of patients with epilepsy to evaluations, which arose with the outbreak of the COVID-19 pandemic. Starting in April 2020, the Clinical Hospital of Psychiatry and Neurology Brasov, where the study took place, was included in the list of national hospitals designated to support the treatment of patients infected with the SARS-CoV-2 virus. The video-EEG monitoring unit, which was situated on the second floor of one of the hospital’s wards, could not be relocated; therefore, its use was restricted when infected patients were admitted to the ward. As a result, patients with epilepsy had their evaluations postponed, and they were unable to go to other public hospitals because the video-EEG unit was the only one in the city. Another limitation was related to conducting the study in a single institution; however, this is offset by the rigor with which it was carried out and the use of the same evaluation standards.

The SSQ is a useful tool for assessing the severity of epileptic seizures from the point of view of the patient and exploring the effects that occur before, during, and after a seizure. A more accurate perception of the phenomena associated with seizures can enable patients to adapt their lifestyle to the type and severity of seizures. QOL is a concept that incorporates physical, psychological, social, and economic aspects, and its assessment should come primarily from the patient through tools provided by physicians or family in order to achieve a balance between the perceived state and the desired state [15]. Suitable instruments such as the SSQ and QOLIE-31-P allow an objective assessment of the daily difficulties faced by patients with epilepsy based on their perception and allow for the identification of the psycho-social consequences of this condition.

The assessment of epilepsy severity is a controversial issue as it is dependent on patient recall, reporting, and witness documentation [35]. SSQ was developed as a multidimensional evaluation of seizures by the patient. Based on the aspects reported by patients and their families, according to which the post-seizure recovery period was the most problematic, the SSQ includes questions covering cognitive, emotional, and physical recovery after such events [14].

The preliminary results of this study show that there is an association between seizure severity and QOL. Seizure-related worry, as measured by the QOLIE-31-P, includes items in the assessment such as concern about a future seizure, fear of possible injuries secondary to a seizure, social embarrassment, and concern about antiepileptic drugs and their effects. The findings reported by patients through the SSQ and QOLIE-31-P scores could be important indicators for helping them and their families improve their perception of seizure severity and QOL.

## 5. Conclusions

The impact of seizures, traditionally studied in terms of frequency and manifestations, can be better understood from the perspective of the patient. Manifestations occurring before, during, and after an epileptic seizure, such as the presence of an aura, involuntary movements, altered state of consciousness, confusion, and affective impairment, explored through the questions of this assessment tool, are relevant data for estimating the consequence of epilepsy on the daily life of the patient. The Romanian version of the SSQ shows good reliability and an adequate translation of content and could be useful in future research measuring seizure severity in more patients with epilepsy from different areas of Romania.

## Figures and Tables

**Table 1:** Sex and clinical characteristics of participants and SSQ TS.

Variables	*n*	Mean (SD) SSQ	*p*	t	r
**Sex**					
Female	38	1.97 (±1.39)	<0.0001	8.717	0.672
Male	29	3.24 (±1.59)	<0.0001	10.93	0.8101
**Seizure frequency**					
1/month	6	3.533 (±1.157)	0.0003	5.363	0.7420
≥2/month	24	4.045 (±1.389)	<0.0001	10.74	0.7150
1–6/year	37	2.441 (±1.227)	<0.0001	7.143	0.4147
**Etiology**					
Structural	40	3.197 (±2.197)	<0.0001	9.947	0.5592
Unknown	24	2.975 (±1.975)	<0.0001	6.496	0.4784
Genetic	3	3.110 (±2.110)	0.2664	1.291	0.2940
**Presence of aura**					
Yes	31	3.741 (±1.323)	<0.0001	11.53	0.6891
No	36	2.574 (±0.9608)	<0.0001	11.02	0.6344
**Number of AED taken**					
Monotherapy	32	2.769 (±1.437)	<0.0001	6.963	0.4388
≥2	25	3.938 (±1.335)	<0.0001	11.0	0.7159
Without	10	2.156 (±0.8904)	<0.0007	4.105	0.4836

**Table 2:** Relationship between SSQ TS and QOLIE-31-P TS and domain score.

SSQ TS vs. *	Mean (±SD)	*p*	t	r
QOLIE-31-P TS	60.06 (±17.45)	<0.0001	26.62	0.843
Energy	26.47 (±25.48)	<0.0001	7.493	0.2984
Mood	30.28 (±25.97)	<0.0001	8.549	0.3564
Daily activities	36.83 (±31.00)	<0.0001	8.892	0.3749
Cognition	38.20 (±31.48)	<0.0001	9.113	0.3862
Medication effects	40.82 (±32.80)	<0.0001	9.402	0.4011
Seizure worry	20.18 (±26.15)	<0.0001	6.572	0.2168
Overall QOL	30.39 (±20.68)	<0.0001	10.77	0.4677

* Pearson.

## Data Availability

The raw data supporting the conclusions of this article will be made available by the authors on request.
